# Energy distribution in an ensemble of nanoparticles and its consequences

**DOI:** 10.3762/bjnano.10.143

**Published:** 2019-07-19

**Authors:** Dieter Vollath

**Affiliations:** 1 NanoConsulting, Primelweg 3, 76297 Stutensee, Germany

**Keywords:** energy distribution, isothermal ensemble, nanoparticle ensemble, normal distribution, particle size distribution, temperature distribution

## Abstract

In general, considerations about isothermal ensembles of nanoparticles assume that each one of the particles is at the same temperature. However, there are experimental indications that such an isothermal ensemble does not exist. Therefore, it is advised to analyze phenomena connected to the temperature distribution within such an ensemble. The detailed analysis presented in this work led to the assumption of a normal distribution of the energy within an ensemble of nanoparticles where basic properties of such an “isothermal” ensemble can be predicted. The width of the energy distribution decreases with increasing particle size. This particle size dependence of the energy per particle controls phase fluctuations in the vicinity of the transformation temperature. Additionally, applying the temperature profile of a phase transformation, it is possible to calculate the particle size distribution of the ensemble with a precision within the scattering range of the experimental data. This is the most important application of this analysis and coincidently a proof of the basic premise. The basic quantity determining the width of the energy distribution is the heat capacity of the particles. For these calculations, bulk data for the heat capacity were successfully applied. This leads to the conclusion that the data for heat capacity of nanoparticles are very close to the bulk values.

## Introduction

General theoretical considerations about ensembles of nanoparticles assume that the ensemble is isothermal. To connect these theoretical considerations with experimental reality, it is necessary to check if such an isothermal ensemble exists at all. For an isothermal ensemble of nanoparticles, one assumes that all the particles are at the same temperature. However, this may not be necessarily true, as there is no information on the temperature of the individual particles. This is of particular relevance in the vicinity of the temperature of a phase transformation, because there are experimental indications of a broad temperature range for the transformation and not, as it is observed in bulk materials, a specific transformation temperature. Within this temperature range, fluctuations are possible [1–2]. However, while these studies give the temperature range where fluctuations may be expected, data about the probability distribution of fluctuations are not presented. This gap needs to be closed. Furthermore, given that the temperature distribution is particle size dependent, the determination of the particle size distribution is inherently possible from the experimentally found temperature dependence of a phase transformation.

## Results and Discussion

### Mathematical model

Maxwell and Boltzmann assumed a normal distribution of the velocity for gas atoms. Following this path, a normal distribution of the energy of nanoparticles was assumed. If the number of particles in an ensemble is *N*, the number of particles at a temperature *T_i_* in the temperature interval Δ*T_i_* is given by *N_i_* = *Nf*(*T_i_*) Δ*T_i_*. The function *f*(*T_i_*) is the probability density function of the temperature. For the distribution of the temperatures *T_i_* of the particle *i* connected to the energy *e_i_* the following distribution law is assumed as:

[1]
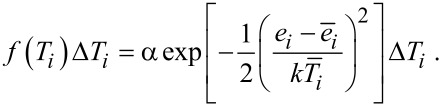


In Equation 1, the quantity 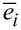 is the average energy of the particles with the mass *m_i_* in an ensemble with the average temperature 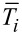
*,* and *k* is the Boltzmann constant. The energies *e_i_* and 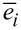 are given by

[2]
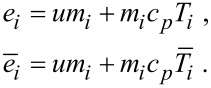


The quantity *u* stands for the enthalpy, and *c_p_* for the heat capacity. As within one ensemble, only one average temperature is possible for the whole ensemble, only one characteristic temperature *T*_char_ is assumed. The characteristic temperature *T*_char_ may be interpreted as the transformation temperature. Inserting the parameters given in Equation 2 in Equation 1 leads to

[3]
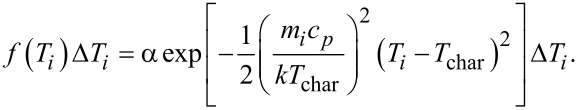


Equation 3 shows that the temperature distribution depends only on the heat capacity and the mass of the individual particles. Even though it reduces generality, for reasons of operability, a spherical geometry was assumed for the calculation of the mass of the particles. The parameter α is determined by the normalization condition

[4]
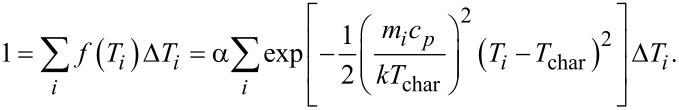


Now, Equation 1 is ready for application. In this context, it is necessary to mention that the application of the Maxwell–Boltzmann energy distribution, as it is observed in gases, leads to an unreasonably broad temperature distribution, making a correlation with experimental data impossible.

### Probability of fluctuations connected to phase transformations

Conventionally, the limiting condition for fluctuations in the vicinity of a phase transformation is [1–2]:

[5]
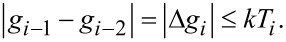


The quantities *g_i_*_−1_ and *g_i_*_−2_ stand for the free enthalpy of the particle *i* in the phases 1 and 2, and |Δ*g_i_*| stands for the difference of the free enthalpy of the two phases at the temperature *T_i_*. Equation 5 gives the boundary condition for fluctuation, but does not give any information about the probability. Therefore, the description of fluctuations has to be expanded.

**Figure 1:**
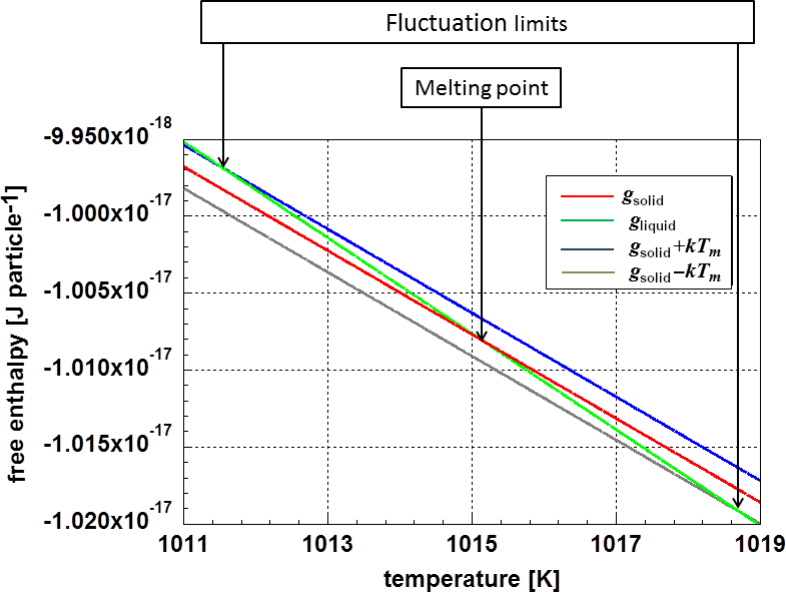
Free enthalpy for a 2 nm gold particle in solid and liquid phase. Additionally, this graph shows the fluctuation limits according to Equation 5.

This necessary extension is demonstrated using as an example of melting gold particles at different particle sizes. Figure 1 displays the free enthalpy of the solid and the liquid phase of a 2 nm gold particle in the vicinity of the melting temperature. Additionally, the data for *g*_solid_ ± *kT* [1–2], describing the limits of the fluctuations, are also given. The fluctuation limits defined by Equation 5 are indicated, too. The necessary thermodynamic data were taken from Arblaster’s review [3]. For the surface energy, in a first approximation, the bulk values were selected [4].

The probability *F*(*T_e_*) of the existence of the solid–liquid phase at the temperature *T_e_* is given by

[6]
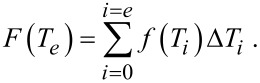


**Figure 2:**
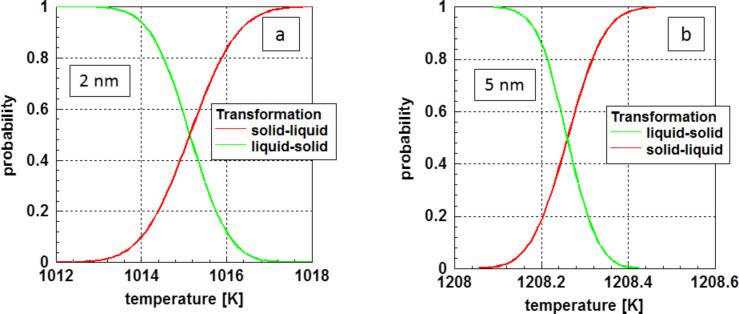
Probability for the transformation of (a) 2 nm and (b) 5 nm diameter gold particles in the vicinity of the melting point.

Figure 2 displays the probability for the solid–liquid phase in the vicinity of the transformation temperature. As examples, particles with a diameter of 2 and 5 nm were selected. One realizes a relatively wide temperature range in the case of the smaller 2 nm particles, whereas this temperature range is very narrow for the 5 nm particles. However, this does not represent experimental reality. As a product of chemical synthesis, one obtains a more or less broad particle size distribution. This widens the actual temperature interval for fluctuations to be observed in an experiment.

The probabilities for the relative amount of the solid and liquid phase given in Figure 2 are equivalent to the probabilities for fluctuations.

### Determination of particle size distribution

One of the major applications of the analysis of the temperature distribution within an ensemble of nanoparticles is the estimation of the size distribution within the ensemble. This is possible in the vicinity of a phase transformation, because according to Equation 3, the temperature distribution depends exponentially on the mass *m_i_*, and therefore, on the diameter *d_i_* of the particles. However, for these calculations, one needs an assumption for the distribution function of particle sizes *g*(*d_i_*). Additionally, to perform detailed calculations, one needs a relation between the transformation temperature and the particle size. As both the range of particle sizes and temperatures are limited, a linear approximation is sufficient. The necessary parameter may be obtained from theory, experimental data, or by a fitting process during the determination of the size distribution.

The starting point for the determination of the particle size distribution is the description of the experimental data by the convolution function.

[7]
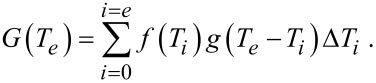


In view of mathematical statistics, the Poisson distribution would be appropriate for particle size distributions stemming from a random process. However, quite often, a normal distribution, which is typical for particles synthesized by chemical precipitation, is applied even when it is known that the tail on the side of large particles is often not properly described. When experimental data is evaluated, the parameters of the distribution functions are determined by a least square fit analysis.

**Figure 3:**
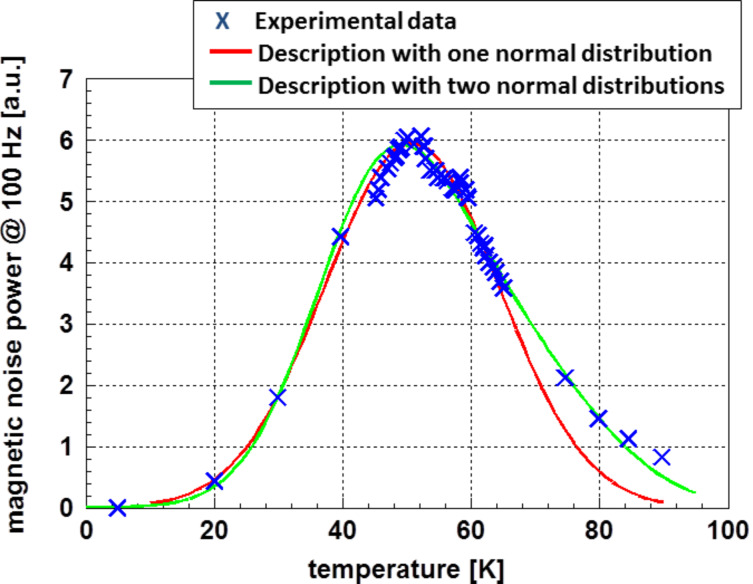
Experimental data for the magnetic noise power of cobalt measured at a frequency of 100 Hz according to Woods et al. [5]. The approximation using Equation 7 was made assuming one normal distribution for the particle sizes (red line) and a modified size distribution taking care of the tail on the side of the larger particles (green line). Using only one normal distribution, the tail on the side of the larger particles is not correctly described. This deviation is minimized by the application of two normal distributions, taking care of the larger particles. The result of the latter approximation is displayed as green line.

**Figure 4:**
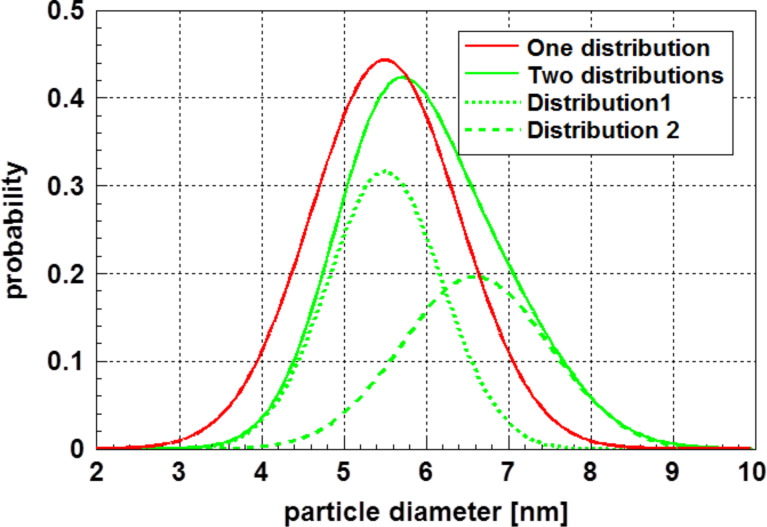
Size distribution of cobalt particles, determined from the transition to superparamagnetic state [5]. Two assumptions were made for the particle size distribution: (1) A normal distribution with the parameters 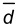 = 5.5 nm and (red curve) σ = 0.85 nm, similar to that assumed by Woods et al. [5] and (2) an extended distribution as a superposition of two normal distributions (green curves) with the parameters 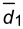 = 5.5 nm and σ_1_ = 0.7 nm. Taking into account the extended tail of the particle size distribution on the side of the larger particles, a second normal distribution with the parameters 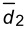 = 6.6 nm and σ_2_ = 0.9 nm was introduced. In both cases, the experimental data given by Woods et al. [5] are well reproduced. However, the assumption of a broader size distribution also takes care of the particles in the tail on the side of the larger particles.

As the first example for determination of a particle size distribution based on the temperature profile of a physical process, the magnetic noise power of cobalt particles at a frequency of 100 Hz (according to Woods et al. [5]) was selected. In this example, 5 nm cobalt particles were scattered on a sheet of silicon oxide. Additionally, these particles were separated by an oleic acid coating of 2 nm. Woods et al. [5] determined the magnetic noise power as a function of temperature during the superparamagnetic transition. For this example, the experimental data obtained from 5 nm particles at a frequency of 100 Hz are displayed in Figure 3. Additionally, in this figure, the approximated data are plotted. The approximation was made in two ways: at first, as given by Woods et al. [5], a normal distribution was selected for the particle size distribution. As it may be clearly seen in Figure 3, this assumption does not describe the course of the magnetic noise power properly. There is significant deviation at higher temperatures, representing larger particles. As fitting using a Weibull or a log-normal distribution did not lead to a satisfying result, an approximation using the sum of two normal distributions was performed. Such a size distribution may occur by minor changes of the conditions during chemical precipitation. As Figure 3 shows, this assumption leads to an improved approximation to the experimental data. The resulting distribution functions for the particle sizes are displayed in Figure 4.

For the calculations leading to the results displayed in Figure 3, the data published by Clusius et al. [6] for the heat capacity of cobalt were applied. Due to the lack of data for nanoparticles, these data were determined based on bulk material. Especially in the temperature region in question for this example, the heat capacity is a highly nonlinear function of temperature. For the relation between the temperature of transformation and particle size, from the experimental data of Woods et al. [5], the relation *d_i_* = 1.83 × 10^−9^ + 6.25 × 10^−11^ *T_i_* was derived. Figure 3 clearly demonstrates that a symmetric size distribution does not fit to the experimental data on the side of the larger particles. The calculations used to derive the parameters describing the particle size distribution presented in Table 1.

**Table 1:** Comparison between the experimental results for the particle size according to Woods et al. [5] and the calculated parameters, determined by the assumption of a normal distribution and an extended distribution of the particle size. The results given for the extended size distribution are obtained from fitting with the sum of two normal distributions. The average particle size was calculated using 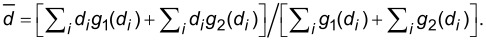

Average particle size			Characteristic temperature
Experimental results [5]	Calculated parameters (normal distribution)	Calculated parameters (extended size distribution)	

5 ± 0.25 nm	5.5 ± 0.76 nm	6.0 ± 0.83 nm	48 K

The comparison displayed in Table 1 demonstrates that the determination of the particle size based solely on the temperature distribution is quite satisfactory; assuming a normal distribution, within the scattering range of the size distributions, the results are identical. The situation is different if one takes care of particles at the tail of the distribution related to the larger particle sizes. However, also in this case, the results may be taken as equal within the scattering range of the particle sizes. Furthermore, one must be aware of the fact that the experimental values obtained for nanoparticle size are, to some extent, dependent on the measurement method. The good fit of the experimental data with the calculated data demonstrates that the values of the heat capacity of nanoparticles may be approximated with high precision data for the equivalent bulk material. This is different for sintered nanomaterials, where, due to the large volume fraction of grain boundaries, the heat capacity is increased [7].

**Figure 5:**
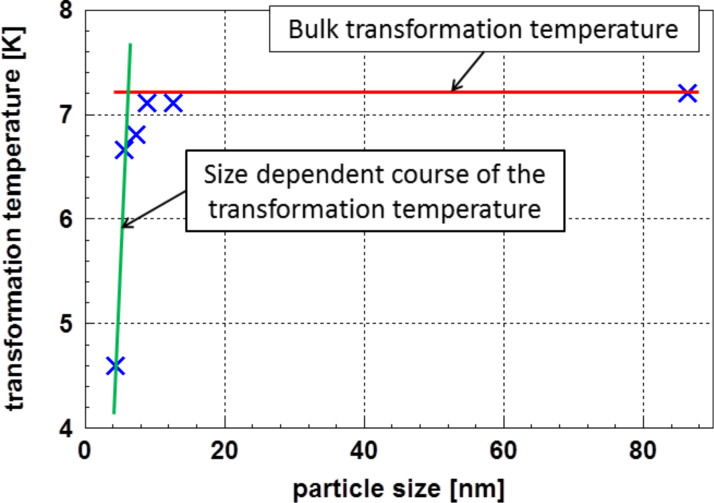
The transformation temperature in lead from normal to superconducting as a function of the particle size according to Li et al. [8]. Below ≈6 nm, a transition to a particle size dependent range is clearly visible.

As a second example, the transition into the superconducting state of nanocrystalline lead is discussed. For these experiments, Li et al. [8] used loosely arranged 4.5 nm particles. The transition from the normal to the superconducting state was determined by measuring the magnetic susceptibility. In bulk lead, the temperature course of this transition is described by the London equation [9]. However, in cases where the particles are smaller than the London penetration depth and the coherence length, the validity of this equation is questionable [8]. Looking at the experimental results of Li et al. [8] one realizes a strong decrease of the transition temperature at particle sizes below ≈6 nm. This course of the transformation temperature as a function of the particle size is displayed in Figure 5.

**Figure 6:**
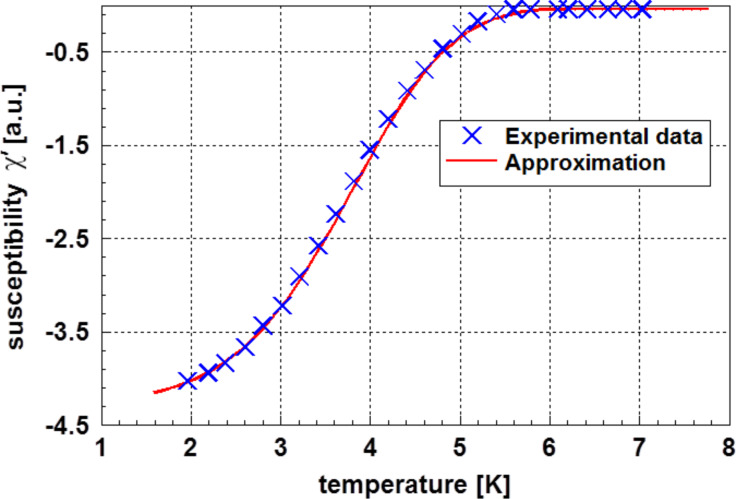
Real part of the magnetic susceptibility χ’ of lead as function of the temperature in the vicinity of the transition to the superconducting state according to Li et al. [8]. Additionally, the approximation according to Equation 7 is shown.

Therefore, the experimental results given for 4.5 nm particles [8] displayed in Figure 5 were used to calculate the particle size distribution according to Equation 7. In Figure 6 the real part of the magnetic susceptibility χ’ is plotted versus the temperature.

**Figure 7:**
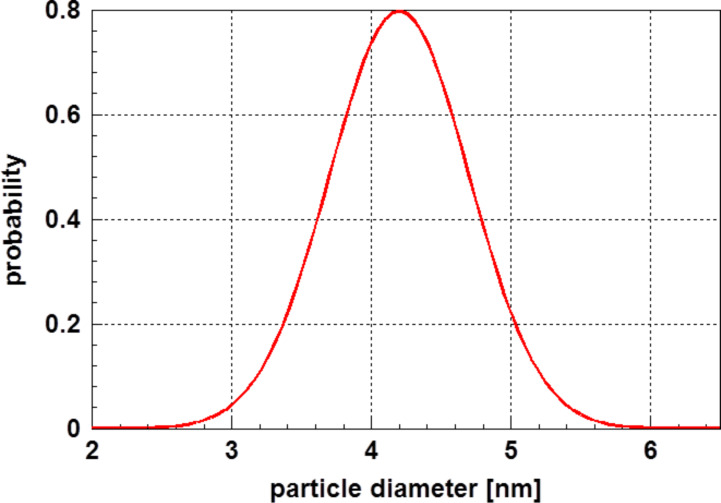
Particle size distribution of lead particles calculated by evaluation of the magnetic susceptibility χ’ as function of the temperature as shown in Figure 6. For this normal distribution, the parameters 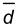 = 4.2 nm and σ = 0.5 nm were determined.

To perform the calculations, for the relation between temperature and particle size, the expression *d_i_* = 1.63 × 10^−9^ + 6.17 × 10^−10^ *T_i_* was derived from Figure 5. For the temperature dependent heat capacity, data published by Horowitz et al. [10] for bulk lead were applied. In contrast to the previous example, in this case not the probability density function (Equation 3) but its integral (the cumulative distribution function) was used. As the scattering of the experimental data was very minor, the approximation is nearly perfect. The results of these calculations are plotted in Figure 6. The evaluation of the experimental data leads to the results displayed in Table 2. The agreement of the calculated particle size with experimental data is satisfying. Because the particle size was determined by evaluation of the X-ray diffraction line broadening in the original paper, Li et al. [8] did not give values for the scattering range of the particle size. From the comparison of the experimental data points with the calculated ones, one can conclude that a normal particle size distribution is an appropriate assumption. Again, it was realized that the bulk values of the heat capacity are sufficiently near to the those of the corresponding nanoparticles to obtain a perfect description. Figure 7 displays the particle size distribution found in this case.

**Table 2:** Comparison between the experimental results for the particle size according to Li et al. [8] and the parameters calculated using Equation 7.

Average particle size		Characteristic temperature
Experimental results [7]	Calculated parameters (normal distribution)	

4.5 nm	4.2 ± 0.5 nm	3.8 K

## Conclusion

The most important result of this study is the fact that, within an “isothermal” ensemble of nanoparticles, there is an individual temperature for each one of the particles. Starting with the assumption that, within an ensemble of nanoparticles, the temperature of the individual particles follows a normal distribution, the probability for fluctuations in the vicinity of a phase transformation was calculated. The results show that this phenomenon is significant only for very small particles. As this phenomenon is particle size dependent, it was also applied to calculate particle size distributions from experimental data of phase transformations. As examples, the superparamagnetic transition of cobalt particles and the transition of lead particles from normal conductance to superconductivity were applied. Within the precision of the experimental determination of particle sizes, the agreement between experimental and calculated data was found to be excellent. Therefore, one may conclude that this evaluation of the temperature dependence of experimental results gives reliable values for the particle size distribution. These successful evaluations confirm the basic premise of this paper: the temperature distribution of particles within an ensemble of nanoparticles follows a normal distribution. In view of experimental reality, the assumption that in an ensemble of nanoparticles each particle is at the same temperature is not realistic. Furthermore, the results show that for loosely arranged nanoparticles, the data for the heat capacity of the bulk material may be applied with sufficient precision.
